# Coronary epicardial and microvascular spasm with transient ischaemic attacks diagnosed by serial spasm provocation

**DOI:** 10.1093/ehjcr/ytaf404

**Published:** 2025-08-21

**Authors:** Hiroyuki Omori, Takuya Makita, Yusuke Miyazaki, Toshiyuki Noda

**Affiliations:** Department of Cardiology, Gifu Prefectural General Medical Center, 4-6-1, Noisshiki, Gifu 500-8717, Japan; Department of Cardiovascular Medicine, Toyohashi Heart Center, 21-1 Gobuto, Toyohashi, Aichi 441-8530, Japan; Department of Clinical Engineering, Gifu Prefectural General Medical Center, 4-6-1 Noisshiki, Gifu 500-8717, Japan; Department of Radiology, Gifu Prefectural General Medical Center, 4-6-1 Noisshiki, Gifu 500-8717, Japan; Department of Cardiology, Gifu Prefectural General Medical Center, 4-6-1, Noisshiki, Gifu 500-8717, Japan

## Physiological concept

Detailed invasive coronary physiology led to the diagnosis of coronary epicardial and microvascular spasm associated with transient ischaemic attacks.

A 50-year-old woman had been experiencing resting chest pain, especially in the early morning, together with stroke-like symptoms—transient loss of consciousness, dysarthria, and left hemiplegia—since 2009. Coronary angiography, brain magnetic resonance imaging (MRI), and magnetic resonance angiography (MRA) performed at several hospitals had been reported as normal.

**Figure 1:**
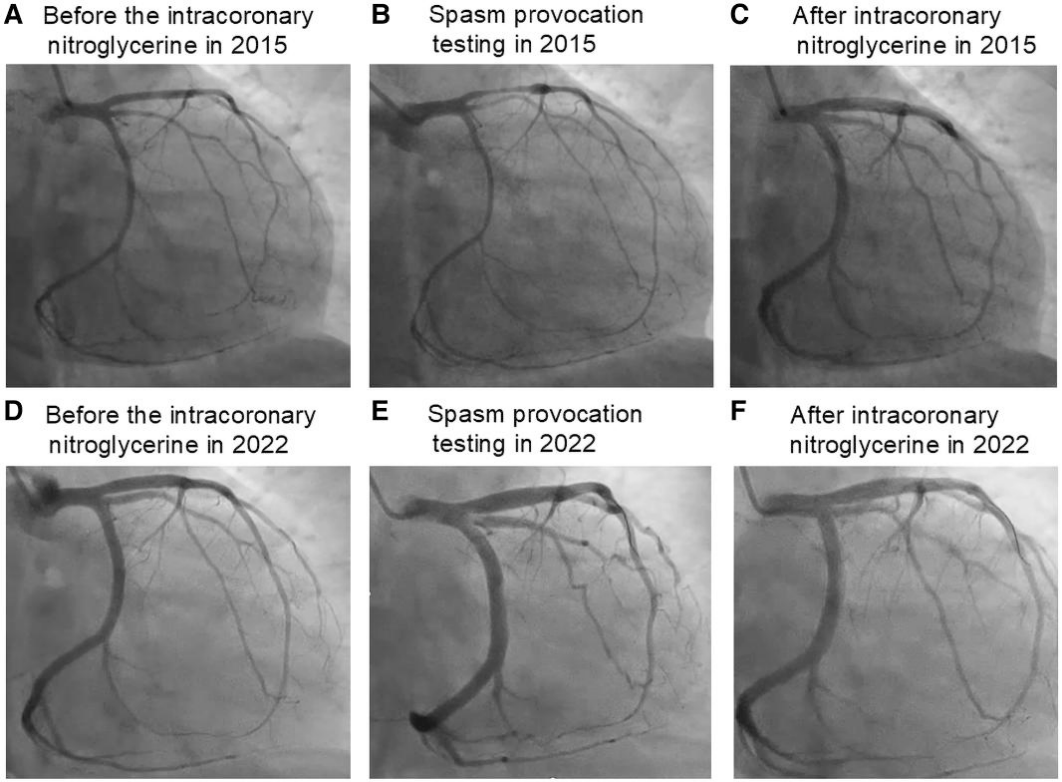
Coronary angiography of the left coronary artery in 2015 and 2022. (*A*) Diffuse spasm before intracoronary nitroglycerin in 2015. (*B*) Ninety-percent luminal narrowing with severe chest pain and stroke-like symptoms during the spasm provocation test in 2015. (*C*) Resolution of luminal narrowing and symptoms after intracoronary nitroglycerin in 2015. (*D*) No obstructive coronary artery disease before intracoronary nitroglycerin in 2022. (*E*) Coronary slow flow without epicardial spasm after spasm provocation in 2022. (*F*) Final angiogram after intracoronary nitroglycerin in 2022.

In 2015, she was referred to our hospital because the symptoms persisted. She had no history of hypertension, dyslipidaemia, diabetes mellitus, arrhythmias, or smoking. High-sensitivity troponin-I concentrations, 12-lead electrocardiography (ECG), and transthoracic echocardiography were unremarkable. Baseline coronary angiography showed moderate, diffuse spasm of the left coronary artery before intracoronary nitroglycerin (*Figure 1A*). A spasm provocation test was performed with intravenous ergonovine (Mochida Pharmaceutical Co.) at 8 µg/min to a cumulative dose of 32 µg over 4 min.^1^ Severe coronary spasm ensued, accompanied by intense chest pain and marked ST-segment depression (*Figure 1B*). Simultaneously, the patient developed the same stroke-like symptoms. All findings resolved completely after 200 µg intracoronary nitroglycerin (*Figure 1C*), and vasospastic angina was diagnosed. Brain MRI and MRA were normal, and a somatoform disorder was considered. Antipsychotics were prescribed, but the symptoms persisted.

**Figure 2:**
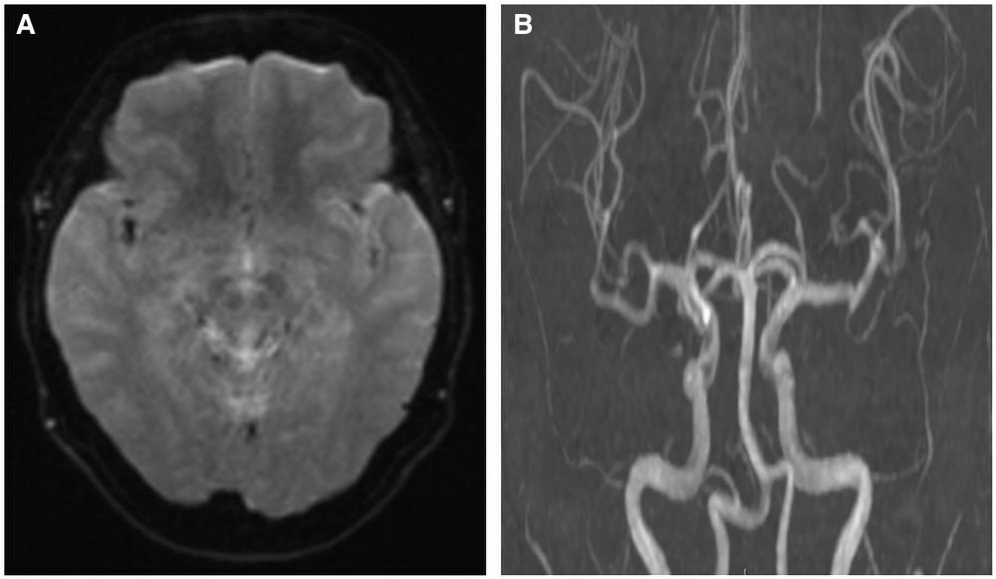
Magnetic resonance imaging and magnetic resonance angiography of the head. (*A*) No abnormalities on diffusion-weighted magnetic resonance imaging. (*B*) Normal magnetic resonance angiography findings.

In 2022, she again presented with worsening symptoms despite oral vasodilators. Repeat angiography showed no obstructive coronary artery disease before nitroglycerin (*Figure 1D*). Coronary microvascular function was evaluated with a pressure-temperature sensor guidewire (PressureWire™ X, Abbott Vascular, St Paul, MN, USA).^2^ Fractional flow reserve was 0.92, coronary flow reserve 6.2, and the index of microcirculatory resistance 9 (see Supplementary material online, *Figure S1*). Intravenous ergometrine was then administered using the 2015 protocol and reproduced chest pain, ST-segment depression in leads V2–V6, and the same stroke-like symptoms. Angiography revealed coronary slow flow without epicardial spasm, consistent with microvascular spasm (*Figure 1E*; Supplementary material online, *Figure S1*). All abnormalities resolved after intracoronary nitroglycerin (*Figure 1F*). Brain MRI and MRA again showed no lesions (*Figure 2*). A neurologist concluded that the reproducible neurological deficits during coronary spasm represented transient ischaemic attacks (TIAs).

Night-time administration of nicorandil 10 mg finally ameliorated both the early-morning angina and the neurological symptoms for the first time in 14 years.^2^

Intravenous ergometrine can provoke vasospasm in any vascular bed; therefore, it may have induced concomitant spasm of the right middle cerebral artery, explaining the transient neurological deficits. Nicorandil, which combines nitrate activity with ATP-sensitive K+ channel opening, improves both epicardial and microvascular spasms and was effective in this patient. Peripheral vascular reactivity has previously been shown to mirror coronary vasomotion, supporting this interpretation.^3^

Serial provocation testing thus established the diagnosis of combined epicardial and microvascular coronary spasm associated with TIAs.

## Supplementary Material

ytaf404_Supplementary_Data

## Data Availability

A data availability statement has been provided, in accordance with the journal’s guidance. All data supporting the findings of this study are available within the article and its Supplementary material.

## Lead author biography

Dr Hiroyuki Omori is an interventional cardiologist from Japan. His fields of interest are coronary physiology, coronary imaging, coronary intervention, and peripheral intervention.

## Supplementary material

**Consent:** The authors confirm that written consent for research purposes was obtained from the patient in compliance with the COPE guidelines.

**Funding:** None declared.
