# Supplementary material for: Coronary epicardial and microvascular spasm with transient ischaemic attacks diagnosed by serial spasm provocation
Source: Eur Heart J Case Rep. 2025 Aug 21;9(9):ytaf404. doi: 10.1093/ehjcr/ytaf404 (PMC12412441; doi:10.1093/ehjcr/ytaf404)

**Supplementary Figure. Assessment with a pressure wire and ergonovine provocation testing of the left coronary artery in 2022**

(A) Baseline angiography showing no obstructive coronary artery disease.

(B) Baseline ECG without ST‑segment changes.

(C) Baseline assessment of microvascular function in the left anterior descending artery; coronary flow reserve (CFR), index of microcirculatory resistance (IMR), and resistive reserve ratio (RRR) were within normal limits.

(D) Coronary slow flow without epicardial spasm after ergonovine provocation.

(E) ST‑segment depression in leads V2–V6 after ergonovine provocation.

(F) Marked elevation of IMR and reductions in CFR and RRR compared with baseline.

Abbreviations: CFR, coronary flow reserve; IMR, index of microcirculatory resistance; RRR, resistive reserve ratio.


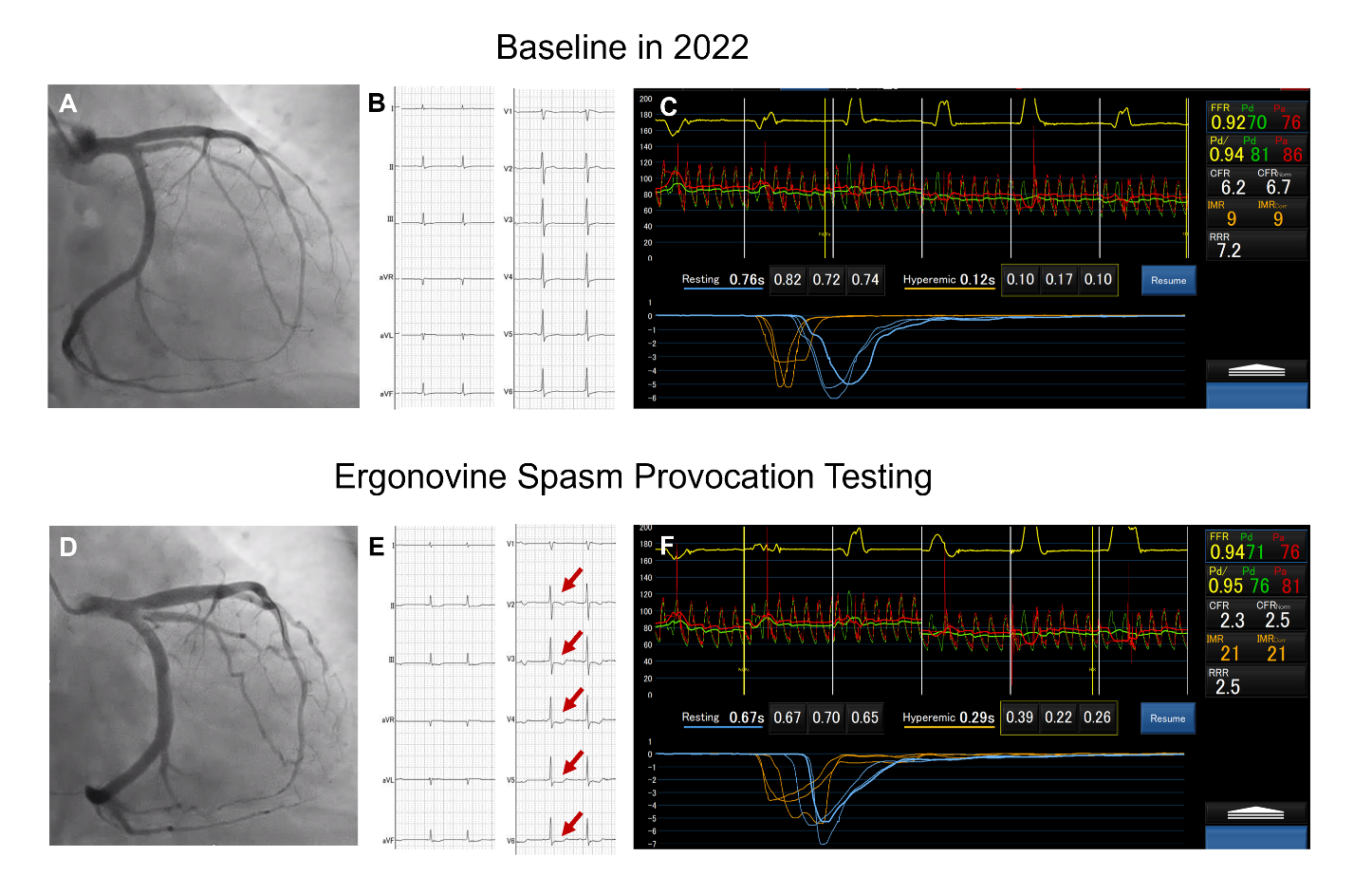

Supplement: ytaf404_Supplementary_Data [file ytaf404_supplementary_data.docx]
